# H3 relaxin inhibits the collagen synthesis *via* ROS‐ and P2X7R‐mediated NLRP3 inflammasome activation in cardiac fibroblasts under high glucose

**DOI:** 10.1111/jcmm.13464

**Published:** 2018-01-05

**Authors:** Xiaohui Zhang, Yu Fu, Hui Li, Li Shen, Qing Chang, Liya Pan, Siting Hong, Xinhua Yin

**Affiliations:** ^1^ The Department of Cardiology The First Affiliated Hospital of Harbin Medical University Harbin China; ^2^ The Department of Cardiology The Fifth hospital of Harbin Harbin China

**Keywords:** cardiac fibrosis, high glucose, NLRP3 inflammasome, P2X7R, ROS

## Abstract

Excessive production of reactive oxygen species (ROS) and P2X7R activation induced by high glucose increases NLRP3 inflammasome activation, which contributes to the pathogenesis of diabetic cardiomyopathy. Although H3 relaxin has been shown to inhibit cardiac fibrosis induced by isoproterenol, the mechanism has not been well studied. Here, we demonstrated that high glucose (HG) induced the collagen synthesis by activation of the NLRP3 inflammasome, leading to caspase‐1 activation, interleukin‐1β (IL‐1β) and IL‐18 secretion in neonatal rat cardiac fibroblasts. Moreover, we used a high‐glucose model with neonatal rat cardiac fibroblasts and showed that the activation of ROS and P2X7R was augmented and that ROS‐ and P2X7R‐mediated NLRP3 inflammasome activation was critical for the collagen synthesis. Inhibition of ROS and P2X7R decreased NLRP3 inflammasome‐mediated collagen synthesis, similar to the effects of H3 relaxin. Furthermore, H3 relaxin reduced the collagen synthesis *via* ROS‐ and P2X7R‐mediated NLRP3 inflammasome activation in response to HG. These results provide a mechanism by which H3 relaxin alleviates NLRP3 inflammasome‐mediated collagen synthesis through the inhibition of ROS and P2X7R under HG conditions and suggest that H3 relaxin represents a potential drug for alleviating cardiac fibrosis in diabetic cardiomyopathy.

## Introduction

Diabetic cardiomyopathy is a microvascular complication of diabetes and the leading cause of end‐stage disease 1. A primary hallmark of diabetic cardiomyopathy is the progressive damage and death of cardiac cells, resulting in cardiac fibrosis. The onset of diabetic cardiomyopathy is insidious, and while the mechanism remains unclear, it is widely accepted that inflammation plays an important role 2. Chronic hyperglycaemia and hyperlipidemia may stimulate the secretion of inflammatory factors, leading to cardiac fibrosis in diabetic cardiomyopathy 3, 4. The NLRP3 (nucleotide‐binding domain and leucine‐rich repeat‐containing family, pyrin domain containing 3) inflammasome is an important component of the innate immune system and is composed of NLRP3, ASC (apoptosis‐associated speck‐like protein containing a CARD) and pro‐caspase‐1. The NLRP3 inflammasome senses endogenous and exogenous danger signals, such as lipopolysaccharides (LPSs) and HG, resulting in the activation of caspase‐1 5. The inflammatory process causes tissue damage that can impair organ function and can result in fibrosis 6. Recently, evidence from clinical and experimental studies supports the hypothesis that the NLRP3 inflammasome is involved in the mechanism of diabetic cardiomyopathy 7. Furthermore, NLRP3 gene silencing has been shown to exert a protective effect against diabetic cardiomyopathy 8.

Functionally, ROS has been proposed to be exclusively involved in the priming step of NLRP3 activation 9. Initially, ROS was shown to be required for NF‐κB activation, which is the priming signal that induces transcription of NLRP3 and pro‐IL‐1β. Additionally, almost all mammalian cells, including cardiac fibroblasts, can release ATP, and extracellular ATP has been implicated in multiple *in vivo* inflammatory responses, including inflammation, fibrosis and tissue damages associated with diabetes. The ionotropic P2X7 receptor (P2X7R) is expressed on cardiac fibroblasts and is activated by extracellular ATP to induce NLRP3 inflammasome assembly as well as the caspase‐1‐dependent processing and release of the pro‐inflammatory cytokines interleukin (IL)‐1β and IL‐18 10. However, ROS and P2X7R expression and their functions in HG‐induced cardiac fibrosis are uncertain.

Relaxin‐3 is an active peptide that plays a protective role in cardiovascular disease. A recent study showed an up‐regulation of myocardial relaxin‐3 during isoproterenol‐induced myocardial ischaemic injury and the protective effect of relaxin‐3 on isoproterenol‐induced cardiac fibrosis, which suggests that relaxin‐3 could be an endogenous cardioprotective factor in ischaemic heart diseases 11. Our recent study found that there were no differences in relaxin‐3 levels between the patients with diabetes and controls 12. However, exogenous H3 relaxin exerts antifibrotic actions *via* RXFP1 and may enhance the collagen inhibitory effects of H2 relaxin 13. Additionally, H3 relaxin inhibited HG‐induced apoptosis in neonatal rat ventricular myocytes by regulating the extrinsic and intrinsic pathways of apoptosis and ERS 14. However, the mechanism behind the antifibrotic actions of H3 relaxin in the heart remains unclear.

Our study investigated the functional role of ROS and P2X7R in neonatal rat cardiac fibroblasts in a HG environment, and whether H3 relaxin inhibits the collagen synthesis through the regulation of the ROS‐ and P2X7R‐mediated NLRP3 inflammasome pathway.

## Materials and methods

### Animals and reagents

Wistar rats (1–3 days old) were purchased from the experimental animal centre in the Second Affiliated Hospital of Harbin Medical University. All animal care and experimental protocols are in accordance with the Animal Management Rule of the Ministry of Health in the People's Republic of China (Document No. 55, 2001) and the Guide for the Care and Use of Laboratory Animals published by the US National Institutes of Health (NIH Publication No. 85‐23, revised 1996). The study was reviewed and approved by the Ethics Committee of The First Affiliated Hospital of Harbin Medical University. Synthetic H3 relaxin was obtained from Phoenix Pharmaceuticals (Belmont, CA, USA), and D‐glucose, hydrogen peroxide, NAC, BzATP and A430879 were purchased from Sigma‐Aldrich (St. Louis, Mo, USA). Antibodies against cleaved caspase‐1 and a‐SMA were purchased from Abcam (Cambridge, UK); Antibodies against type I collagen, type III collagen and NLRP3 were purchased from Bioss (Beijing, China). Antibodies against IL‐1β and IL‐18 were purchased from Novus Biologicals (CO, USA). Other chemicals and reagents were of analytical grade.

### Primary neonatal rat cardiac fibroblasts culture

The ventricles of Wistar neonatal rats (1–3 days old) regardless of sex were isolated and minced into 1‐ to 2‐mm pieces with scissors, and the cells were dissociated by incubating in 0.25% trypsin for 2–3 min. between 20–25 times. The supernatant from the first digestion was discarded, and the cells from the subsequent digestions were placed in DMEM supplemented with 10% calf serum and centrifuged. The cells were resuspended in DMEM with 10% calf serum, and the cells were plated into a culture bottle for 1.5 hrs (37°C in a 5% CO2 incubator) for purification. Then, the suspension was discarded, and the sediment (cardiac fibroblasts) was maintained and cultured in an incubator (37°C with 5% CO2). Cell experiments were divided into three parts: in the first experiment, cardiac fibroblasts were incubated in media containing normal glucose (NG, 5.5 mmol/l), HG (33 mmol/l) and HG+H3 relaxin (100 ng/ml) for 48 hrs; in the second experiment, cardiac fibroblasts were incubated in media containing NG, HG (33 mmol/l), HG +H3 relaxin (100 ng/ml), HG+H_2_O_2_ (1 μmol/l, ROS agonist), HG+NAC (10 mmol/l, ROS antagonist) and HG+H_2_O_2_+H3 relaxin for 48 hrs; and in the third experiment, cardiac fibroblasts were incubated in media containing NG, HG (33 mmol/l), HG+H3 relaxin (100 ng/ml), HG+BzATP (100 mmol/l, P2X7R agonist), HG +A438079 (10 mmol/l, P2X7R antagonist) and HG+BzATP+H3 relaxin for 48 hrs.

### Detection of intracellular ROS

Cardiac fibroblasts were incubated with 10 μM of 5‐(and 6)‐chloromethyl‐2′,7′ ‐dichlorodihydrofluorescein diacetate, acetyl ester (Beyotime, China) in the dark for 15 min. at 37°C and the fluorescence intensity was detected by a microplate reader (SpectraMax M5, Molecular Devices, US) using 488 nm excitation and 525 nm emission wavelengths.

### Western blot analyses

Cardiac fibroblasts were washed with PBS and resuspended in cold lysis buffer containing phenylmethylsulfonyl fluoride (PMSF). The cell lysate was incubated on ice for 30 min. and centrifuged at 12,000 × *g* for 10 min. at 4°C. The protein concentration in the supernatant was determined using a BCA‐200 protein assay kit (Beyotime, China). Equal amounts of protein (20 μg) were separated by 12% SDS‐PAGE and transferred to a PVDF membrane. The non‐specific proteins were blocked by incubating the membrane with 5% non‐fat dry milk for 1 hr at room temperature with agitation. The membrane was then incubated overnight at 4°C with the following primary antibodies: anti‐β‐actin (1:1000), anti‐NLRP3 (1:1000), anti‐cleaved caspase‐1 (1:1000), anti‐IL‐1β (1:1000), anti‐IL‐18 (1:1000), anti‐P2X7R (1:1000), anti‐a‐SMA (1:1000), anti‐I‐collagen (1:1000) and anti‐III‐collagen (1:1000). The membrane was then washed three times for 10 min. each followed by incubation with the appropriate corresponding fluorescently labelled secondary IgG antibody. Antigen–antibody complexes were visualized using the Odyssey Infrared Imaging System and Odyssey v3.0 software. The levels of proteins were normalized to the level of β‐actin.

### Detection of cytokines in culture media

The culture media was centrifuged for 10 min. at 1000 × *g*. IL‐1β and IL‐18 were detected using a Milliplex MAP Rat Cytokine/Chemokine Magnetic Bead Panel kit (Cat. RECYTMAG‐65K, EMD Millipore, Darmstadt, Germany).

### Statistical analyses

Each experiment was repeated a minimum of six times, and the results were expressed as the mean ± S.D. The GraphPad Prism 5.0 software was used for data analyses. For comparisons of more than two groups, we used anova followed by a Newman–Keuls multiple comparisons test. *P* < 0.05 was considered statistically significant.

## Results

### H3 relaxin inhibited the collagen synthesis induced by HG

**Figure 1:**
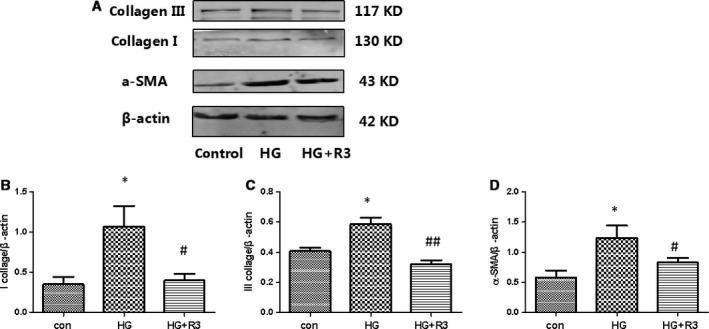
H3 relaxin inhibited the collagen synthesis induced by HG. (**A**) Collagen I, collagen III and a‐SMA protein expressions were analysed by Western blot. (**B**) The protein levels of collagen I were normalized to β‐actin (collagen I/β‐actin). (**C**) The protein levels of collagen III were normalized to β‐actin (collagen III/β‐actin). (**D**) The protein levels of a‐SMA were normalized to β‐actin (a‐SMA/β‐actin). Data are the means ± S.D., and each measurement carried out six times. *P < 0.05 *versus* control, **P < 0.01 versus control, ^#^ *P* < 0.05 *versus* HG, ^##^ *P* < 0.01 *versus* HG.

To investigate the functional effects of H3 relaxin on HG‐induced cardiac fibrosis, neonatal rat cardiac fibroblasts were pre‐treated with H3 relaxin (100 ng/ml) for 30 min. and then exposed to HG (33 mmol/ml) for 48 hrs, whereas the control group was treated with NG (5.5 mmol/ml). We demonstrated that treatment decreased the HG‐induced expression of collagen type I and III protein, which was assessed using Western blot (Fig. 1). Additionally, H3 relaxin administration decreased the HG‐induced protein expression of a‐SMA, a fibroblast phenotype transition marker.

### H3 relaxin inhibited the collagen synthesis by regulating NLRP3 inflammasome activation

**Figure 2:**
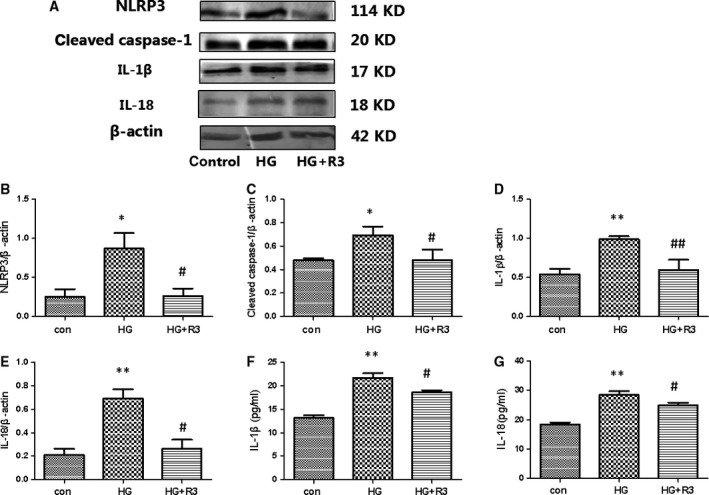
H3 relaxin inhibited NLRP3 inflammasome activation in cardiac fibroblasts. (**A**) The protein expression of NLRP3 inflammasome markers (NLRP3, cleaved caspase‐1, IL‐1β and IL‐18) was analysed by Western blot. (**B**) The protein levels of NLRP3 were normalized to β‐actin (NLRP3/β‐actin). (**C**) The protein levels of cleaved caspase‐1 were normalized to β‐actin (cleaved caspase‐1/β‐actin). (**D**) The protein levels of IL‐1β were normalized to β‐actin (IL‐1β/β‐actin). (**E**) The protein levels of IL‐18 were normalized to β‐actin (IL‐18/β‐actin). (**F**) The protein levels of IL‐1β in cell culture media. (**G**) The protein levels of IL‐18 in cell culture media. Data are the means ± S.D., and each measurement carried out six times. *P < 0.05 *versus* control, **P < 0.01 *versus* control, ^#^P < 0.05 *versus* HG, ^##^ *P* < 0.01 *versus* HG.

As NLRP3 inflammasome activation was involved in HG‐induced cardiac fibrosis in cardiac fibroblasts, HG could induce the collagen synthesis through a rapid accumulation of IL‐18 and IL‐1β. We investigated the protein levels of NLRP3, cleaved caspase‐1, IL‐18 and IL‐1β in HG‐treated cardiac fibroblasts. H3 relaxin suppressed the HG‐mediated increases in NLRP3, cleaved caspase‐1, IL‐18 and IL‐1β protein levels (Fig. 2). To investigate the functional effects of H3 relaxin on HG‐induced NLRP3 activation, we measured the levels of IL‐18 and IL‐1β in culture media (Fig. 2). The levels of IL‐18 and IL‐1β in culture media were significantly higher in the HG group than in the NG group (*P* < 0.01). Moreover, the levels of IL‐18 and IL‐1β were significantly lower in the HG+R3 group than in the HG group (*P* < 0.05). These data suggest that HG‐induced collagen synthesis is at least partially mediated by NLRP3 inflammasome pathway activation and may be inhibited by H3 relaxin.

### H3 relaxin inhibited the collagen synthesis *via* ROS‐mediated NLRP3 inflammasome activation

**Figure 3:**
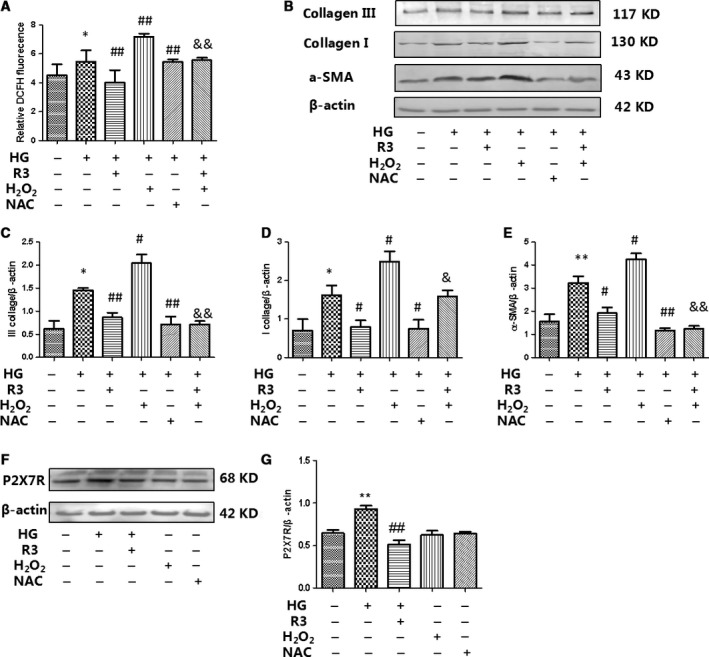
H3 relaxin inhibited the collagen synthesis *via* ROS activation. (**A**) ROS levels in cardiac fibroblasts were analysed by DCFH. (**B**) Collagen I, collagen III and a‐SMA protein expressions were analysed by Western blot. (**C**) The protein levels of collagen I were normalized to β‐actin (collagen I/β‐actin). (**D**) The protein levels of collagen III were normalized to β‐actin (collagen III/β‐actin). (**E**) The protein levels of a‐SMA were normalized to β‐actin (a‐SMA/β‐actin). (**F**) P2X7R protein expressions were analysed by Western blot. (**G**) The protein levels of P2X7R were normalized to β‐actin (P2X7R/β‐actin). Data are the means ± S.D., and each measurement carried out six times. *P < 0.05 *versus* control, **P < 0.01 *versus* control, ^#^ *P* < 0.05 *versus* HG, ^##^ *P* < 0.01 *versus* HG, ^&^ *P* < 0.05 *versus* HG+H_2_O_2_, ^&&^ *P* < 0.01 *versus* HG+H_2_O_2_.

It is generally accepted that the NLRP3 inflammasome is activated by cellular oxidative stress, such as H_2_O_2_. The generation of reactive oxygen species (ROS) is considered to be critical for NLRP3 inflammasome activation. We found that HG increased the level of ROS; however, compared to the HG group, the ROS levels were decreased in the H3 relaxin group. Consistently, ROS levels were increased by H_2_O_2_ treatment and decreased by NAC treatment (Fig. 3).

To determine whether H3 relaxin inhibited cardiac fibrosis by regulating ROS‐induced NLRP3 inflammasome activation, we first treated cardiac fibroblasts with HG with/without ROS agonist (H_2_O_2_) or inhibitor (NAC). We observed that H_2_O_2_ aggravated HG‐induced increases in collagen type I and III as well as a‐SMA protein expression, and NAC alleviated HG‐induced increases in collagen type I and III as well as a‐SMA protein expression, similar to H3 relaxin treatment (Fig. 3A–E). To determine whether ROS agonist (H_2_O_2_) or inhibitor (NAC) affected the function of P2X7R, we observed P2X7R expression by Western blot. We found that ROS agonist (H_2_O_2_) or inhibitor (NAC) does not affect P2X7R expression (Fig. 3F and G).

**Figure 4:**
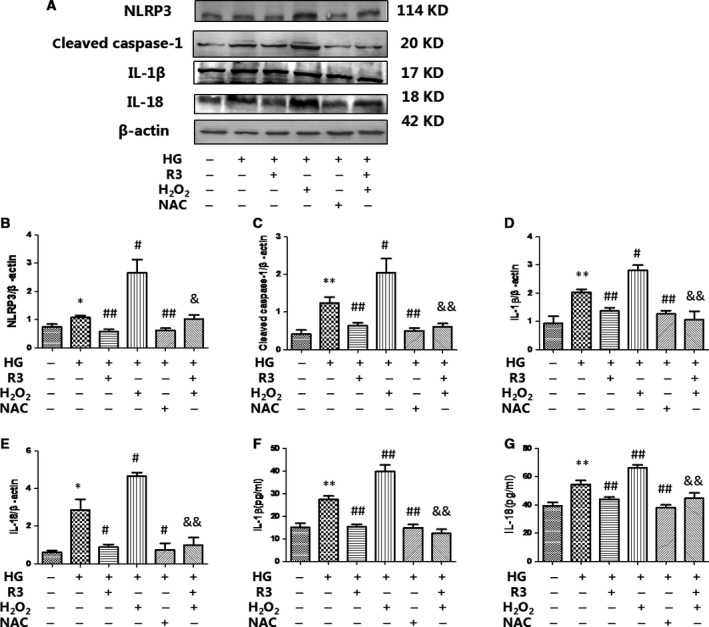
H3 relaxin inhibited ROS‐mediated NLRP3 inflammasome activation. (**A**) The protein expression of NLRP3 inflammasome markers (NLRP3, cleaved caspase‐1, IL‐1β and IL‐18) was analysed by Western blot. (**B**) The protein levels of NLRP3 were normalized to β‐actin (NLRP3/β‐actin). (**C**) The protein levels of cleaved caspase‐1 were normalized to β‐actin (cleaved caspase‐1/β‐actin). (**D**) The protein levels of IL‐1β were normalized to β‐actin (IL‐1β/β‐actin). (**E**) The protein levels of IL‐18 were normalized to β‐actin (IL‐18/β‐actin). (**F**)The protein levels of IL‐1β in cell culture media. (**G**) The protein levels of IL‐18 in cell culture media. Data are the means ± S.D., and each measurement carried out six times. *P < 0.05 *versus* control, **P < 0.01 *versus* control, ^#^P < 0.05 *versus* HG, ^##^ *P* < 0.01 *versus* HG, ^&^ *P* < 0.05 *versus* HG+H_2_O_2_, ^&&^ *P* < 0.01 *versus* HG+H_2_O_2_.

We also demonstrated that H_2_O_2_ stimulation amplified intracellular HG‐induced NLRP3 inflammasome protein (NLRP3 and cleaved caspase‐1) expression and activation (IL‐18 and IL‐1β) in cardiac fibroblasts, whereas these effects were attenuated by H3 relaxin (Fig. 4). Meanwhile, we further observed that pre‐treatment with NAC could also markedly inhibit HG‐enhanced NLRP3 inflammasome expression and activation, indicating that H3 relaxin inhibits ROS‐induced NLRP3 inflammasome activation in HG‐induced collagen synthesis. Consistently, the levels of IL‐18 and IL‐1β in the culture media were significantly higher in the HG+H_2_O_2_ group than in the HG group; moreover, similar to the H3 relaxin group, the levels of IL‐18 and IL‐1β were significantly lower in the HG+NAC group than in the HG group, (*P* < 0.05). These data suggest that HG‐induced collagen synthesis is at least partially mediated by ROS‐induced NLRP3 inflammasome pathway activation, which may be inhibited by H3 relaxin.

### H3 relaxin inhibited the collagen synthesis *via* P2X7R‐mediated NLRP3 inflammasome activation

**Figure 5:**
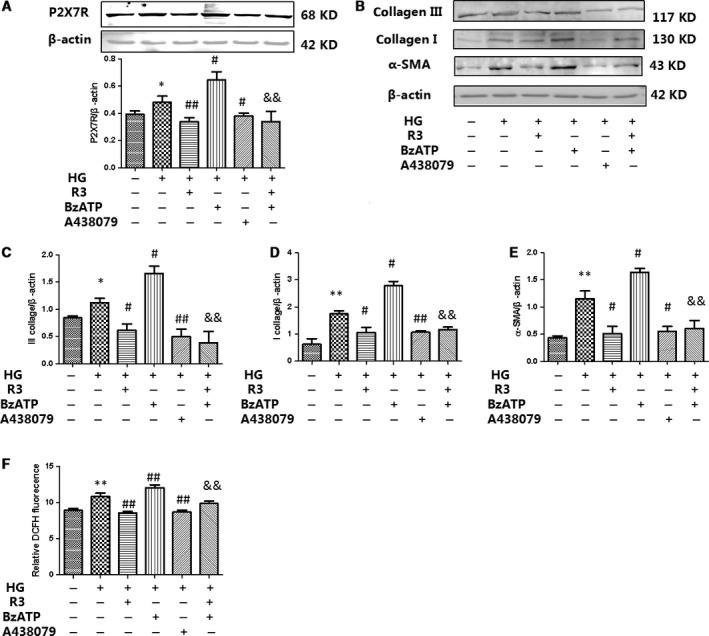
H3 relaxin inhibited the collagen synthesis *via* P2X7R activation. (**A**) P2X7R protein expressions were analysed by Western blot. (**B**) Collagen I, collagen III and a‐SMA protein expressions were analysed by Western blot. (**C**) The protein levels of collagen III were normalized to β‐actin (collagen III/β‐actin). (**D**) The protein levels of collagen I were normalized to β‐actin (collagen I/β‐actin). (**E**) The protein levels of a‐SMA were normalized to β‐actin (a‐SMA/β‐actin).(**F**) ROS levels in cardiac fibroblasts were analysed by DCFH. Data are the means ± S.D., and each measurement carried out six times. *P < 0.05 *versus* control, **P < 0.01 *versus* control, ^#^ *P* < 0.05 *versus* HG, ^##^ *P* < 0.01 *versus* HG, ^&^ *P* < 0.05 *versus* HG+BzATP, ^&&^ *P* < 0.01 *versus* HG+BzATP.

Numerous studies have suggested that potassium (K+) efflux is a necessary signal upstream of NLRP3 activation. It is known that extracellular ATP engages the ATP‐gated cation channel P2X7R whereas bacterial toxins cause membrane pore formation to trigger K+ efflux. We found that the HG‐induced increase in P2X7R expression was inhibited by H3 relaxin (Fig. 5); however, the role of P2X7R in the H3 relaxin‐mediated inhibition of the collagen synthesis has yet to be characterized. We first treated cardiac fibroblasts with HG with/without P2X7R agonist (BzATP) or inhibitor (A438079). We observed that BzATP aggravated HG‐induced increases in protein expression of collagen type I, collagen type III and a‐SMA, and similar to H3 relaxin, A438079 alleviated HG‐induced increases in protein expression of collagen type I, collagen type III and α‐SMA (Fig. 5). In addition, we found that HG induces ROS production in neonatal rat cardiac fibroblasts, an effect eliminated by H3 relaxin and P2X7R antagonist A438079. A similar effect was observed with a P2X7R agonist (BzATP), P2X7R activation with BzATP induces ROS production in neonatal rat cardiac fibroblasts (Fig. 3F and G). These results indicate that ROS increases induced by BzATP occur through P2X7R activation.

**Figure 6:**
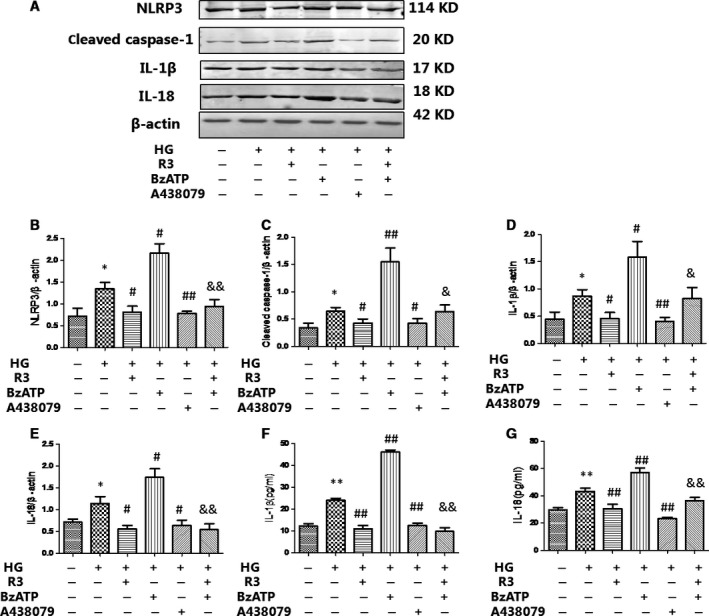
H3 relaxin inhibited P2X7R‐mediated NLRP3 inflammasome activation (**A**) The protein expression of NLRP3 inflammasome markers (NLRP3, cleaved caspase‐1, IL‐1β and IL‐18) was analysed by Western blot. (**B**) The protein levels of NLRP3 were normalized to β‐actin (NLRP3/β‐actin). (**C**) The protein levels of cleaved caspase‐1 were normalized to β‐actin (cleaved caspase‐1/β‐actin). (**D**) The protein levels of IL‐1β were normalized to β‐actin (IL‐1β/β‐actin). (**E**) The protein levels of IL‐18 were normalized to β‐actin (IL‐18/β‐actin). (**F**) The protein levels of IL‐1β in cell culture media. (**G**) The protein levels of IL‐18 in cell culture media. Data are the means ± S.D., and each measurement carried out six times. *P < 0.05 *versus* control, **P < 0.01 *versus* control, ^#^ *P* < 0.05 *versus* HG, ^##^ *P* < 0.01 *versus* HG, ^&^ *P* < 0.05 *versus* HG+BzATP, ^&&^ *P* < 0.01 *versus* HG+BzATP.

We also demonstrated that P2X7R stimulation amplified intracellular HG‐induced NLRP3 inflammasome protein (NLRP3 and cleaved caspase‐1) expression and activation (IL‐18 and IL‐1β) in cardiac fibroblasts, whereas these effects were attenuated by H3 relaxin (Fig. 6). Meanwhile, we further observed that pre‐treatment with A438079 could markedly inhibit HG‐enhanced NLRP3 expression and activation, indicating that H3 relaxin inhibited P2X7R‐induced NLRP3 inflammasome activation in HG‐induced collagen synthesis. Consistently, the levels of IL‐18 and IL‐1β in culture media were significantly higher in the HG+BzATP group than in the HG group; moreover, similar to H3 relaxin treatment, the levels of IL‐18 and IL‐1β were significantly lower in the HG+A438079 group than in the HG group (*P* < 0.05). These data suggest that HG‐induced collagen synthesis is at least partially mediated by P2X7R‐induced NLRP3 inflammasome pathway activation, which may be inhibited by H3 relaxin.

## Discussion

The present study aimed to investigate the roles of ROS and P2X7R and their physiological contributions to cardiac fibrosis *via* NLRP3 inflammasome activation under HG conditions in neonatal rat cardiac fibroblasts, which is inhibited by H3 relaxin. Here, we showed that HG‐augmented NLRP3 inflammasome activation and induced the collagen synthesis by increasing IL‐18 and IL‐1β production *via* ROS and P2X7R signalling. In addition, we observed that H3 relaxin directly inhibited HG‐induced cardiac fibrosis *via* decreasing ROS‐ and P2X7R‐dependent NLRP3 inflammasome activation in cardiac fibroblasts.

The contribution of HG to inflammatory processes was considered a major risk factor for the development of diabetes and cardiovascular complications 15. Studies have reported that HG‐induced oxidative stress elevated ROS and activated P2X7R, which were identified as activators of the NLRP3 inflammasome. P2X7R, which contributes to the activation of the NLRP3 inflammasome, is an oxidative stress and metabolic sensor 16, 17. In this study, our observations further demonstrated that H3 relaxin attenuated the enhancement of ROS‐ and P2X7R‐mediated NLRP3 inflammasome activation by HG, indicating that H3 relaxin inhibited HG‐induced collagen synthesis *via* ROS‐ and P2X7R‐mediated NLRP3 inflammasome activation.

Recently, ROS was identified as a key factor that linked oxidative stress to NLRP3 inflammasome activation; thus, we examined whether ROS could mediate HG‐induced NLRP3 inflammasome activation in cardiac fibroblasts inhibited by H3 relaxin. A study reported that the NLRP3 inflammasome contributed to the development of diabetic cardiomyopathy *via* ROS‐induced caspase‐1 and IL‐1β activation, which are the effectors of the NLRP3 inflammasome. Indeed, HG could induce IL‐1β and IL‐18 secretion in cardiac fibroblasts, and we further demonstrated that it was dependent on NLRP3 inflammasome activation. Moreover, we found that ROS production *via* NLRP3 inflammasome activation could act as a critical regulator of HG‐induced cardiac fibrosis. ROS was necessary to trigger NLRP3 inflammasome activation due to its regulation of NLRP3 assembly and mediation of IL‐1β and IL‐18 production. The NLRP3 inflammasome is a protein complex that consists of an NLRP3 inflammasome sensor, the adaptor protein ASC and caspase‐1. Our results confirmed that ROS directly regulated the caspase‐1/ASC inflammasome complex under HG conditions. Moreover, our data suggest that ROS‐ and HG‐induced NLRP3 inflammasome activation are inhibited by H3 relaxin. Although the mechanism by which H3 relaxin inhibits cardiac fibrosis in cardiac fibroblasts is unknown, a study suggested that H3 relaxin protects astrocytes from ischaemic conditions through the reduction in ROS production and the maintenance of mitochondrial membrane potential 18. Our study suggested that H3 relaxin reduced oxidative stress and lowered NLRP3 inflammasome expression as well as activation in HG conditions, which supports the hypothesis that H3 relaxin might inhibit cardiac fibrosis in HG conditions.

P2X7R was critical for mediating NLRP3 inflammasome activation and could act as a sensor to modulate the levels of redox signalling molecules 19. A study suggested that myocardial fibrosis in myocarditis is caused by fibroblast migration initiated *via* the activation of the P2X7R‐MAPK signalling pathway 20. P2X7R was also implicated in the pathogenesis of type 1 diabetes 21, 22, 23, 24, 25, 26, 27. A previous study showed that the absence of the P2X7R provided resistance against the induction of diabetes and suggested that P2X7R‐targeted therapy may be useful against clinical diabetes 28. Our results demonstrated that HG triggered the activation of P2X7R and subsequently NLRP3 inflammasome activation in cardiac fibroblasts, and we found that H3 relaxin inhibited P2X7R activation under HG conditions, indicating that H3 relaxin regulates NLRP3 inflammasome activation through P2X7R under HG conditions. Our results demonstrated that the inhibition of P2X7R markedly reduced HG‐up‐regulated NLRP3 inflammasome expression and activation, suggesting that the initiation of NLRP3 inflammasome activation under HG conditions is dependent on HG‐mediated P2X7R activation. Therefore, our results indicated that HG promotes NLRP3 inflammasome‐dependent cardiac fibrosis through the P2X7R pathway in rat cardiac fibroblasts. In this study, we treated cardiac fibroblasts with HG for 48 hrs to investigate the underlying mechanisms of the NLRP3 inflammasome in the progression of diabetes. Recent studies have shown that NLRP3 inflammasome activation is elevated in cardiac fibroblasts from diabetic rats. Hence, we elucidated a novel mechanism in which P2X7R‐mediated NLRP3 inflammasome activation is inhibited by H3 relaxin in the pathogenesis of diabetes. Recent study found that neuronal P2X7R activation leads to ROS production and subsequent nociceptive pain in mice. Together, the data indicate that P2X7R‐induced ROS play a critical role in the P2X7R signalling pathway of the central nervous system 29. Another study found that Abeta‐stimulated ROS generation in microglial cells is regulated by ATP released from the microglia in an autocrine manner 30. In our study, we found that ROS increased by BzATP occur through P2X7R activation and decreased by P2X7R inhibitor, and ROS agonist (H_2_O_2_) or inhibitor (NAC) does not affect P2X7R expression. Consistent to recent studies, generation of ROS following P2X7R activation is well established in other cell types. Here, we provide evidence that P2X7R activation leads to ROS production and subsequent inflammation in HG‐induced collagen synthesis and H3 relaxin inhibited P2X7R activation mediated ROS production in neonatal rat cardiac fibroblasts induced by HG.

In conclusion, we demonstrated that H3 relaxin inhibited HG‐induced the collagen synthesis through ROS‐ and P2X7R‐mediated NLRP3 inflammasome activation in neonatal rat cardiac fibroblasts. Therefore, our results implicate H3 relaxin as a potential therapeutic strategy for ameliorating NLRP3 inflammasome activation related to HG‐induced cardiac fibrosis in diabetes.

## Conflict of interest

On behalf of all authors, the corresponding author states that there is no conflict of interests.
