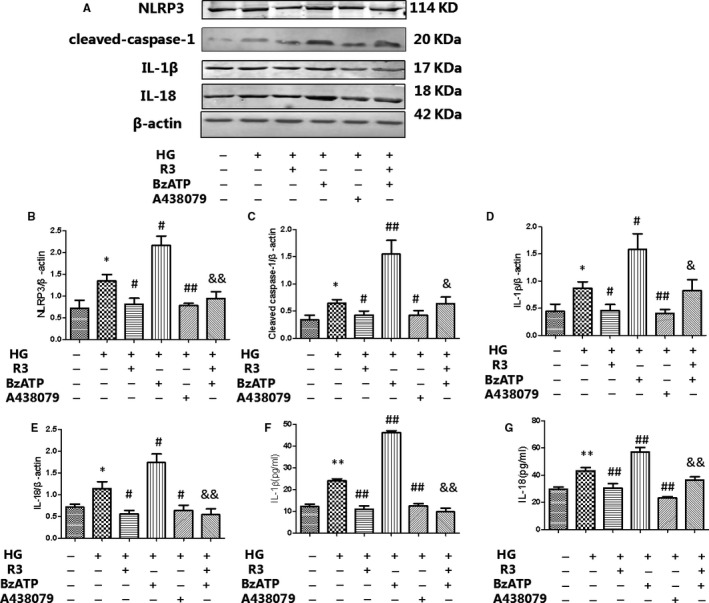# Corrigendum: H3 relaxin inhibits the collagen synthesis via ROS‐ and P2X7R‐mediated NLRP3 inflammasome activation in cardiac fibroblasts under high glucose

**DOI:** 10.1111/jcmm.13615

**Published:** 2018-06

**Authors:** Xinhua Yin

**Affiliations:** ^1^ The Department of Cardiology The First Affiliated Hospital of Harbin Medical University Harbin China

We found a mistake when we read our article. In figure 6, the band of cleaved caspase‐1 was not about cleaved caspase‐1. The incorrect figure 6

should be replaced with the revised and corrected figure 6.

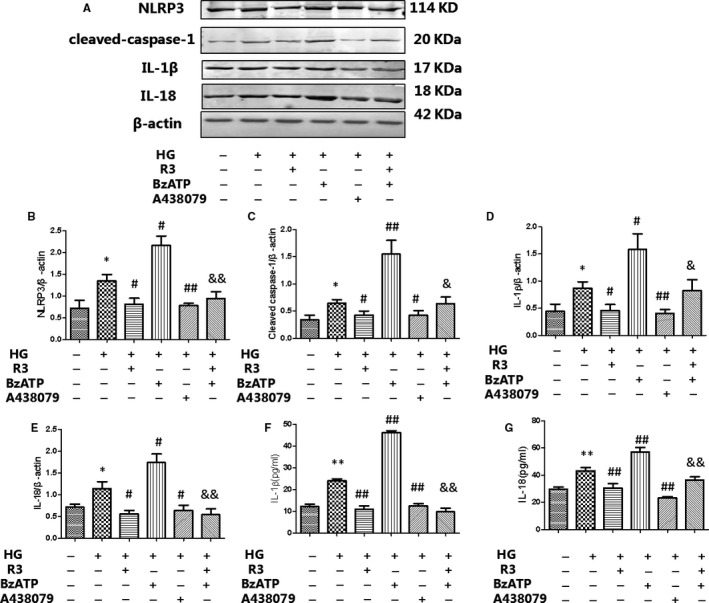

should be replaced with the revised and corrected figure 6.